# Ileal immune tonus is a prognosis marker of proximal colon cancer in mice and patients

**DOI:** 10.1038/s41418-020-00684-w

**Published:** 2020-12-01

**Authors:** Marion Picard, Satoru Yonekura, Karolina Slowicka, Ioanna Petta, Conrad Rauber, Bertrand Routy, Corentin Richard, Valerio Iebba, Maryam Tidjani Alou, Sonia Becharef, Pierre Ly, Eugenie Pizzato, Christian H. K. Lehmann, Lukas Amon, Christophe Klein, Paule Opolon, Ivo Gomperts Boneca, Jean-Yves Scoazec, Antoine Hollebecque, David Malka, François Ghiringhelli, Diana Dudziak, Geert Berx, Lars Vereecke, Geert van Loo, Guido Kroemer, Laurence Zitvogel, Maria Paula Roberti

**Affiliations:** 1 Gustave Roussy Cancer Campus (GRCC), Villejuif, France; 2 Institut National de la Santé Et de la Recherche Médicale (INSERM) U1015, Equipe Labellisée-Ligue Nationale contre le Cancer, Villejuif, France; 3 Institut Pasteur, Unit Biology and Genetics of the Bacterial Cell Wall, Paris, France; 4 INSERM, Equipe Avenir, Paris, France; 5 Université Paris-Saclay, Le Kremlin-Bicêtre cedex, France; 6 VIB Center for Inflammation Research, Ghent, Belgium; 7 Department of Biomedical Molecular Biology, Ghent University, Ghent, Belgium; 8 Department of Internal Medicine and Pediatrics, Ghent University, Ghent, Belgium; 9 Department of Medical Oncology, Center GF Leclerc, Dijon, France; 10 Plateform Transfer in Biological Oncology, Dijon, France; 11 Department of Medical, Surgical and Health Sciences, University of Trieste, Trieste, Italy; 12 Department of Dermatology, Laboratory of Dendritic Cell Biology, Medical Immunology Campus Erlangen, University Hospital of Erlangen, Friedrich-Alexander-University (FAU) of Erlangen-Nürnberg, Erlangen, Germany; 13 Equipe Labellisée par la Ligue Contre le Cancer, Université de Paris, Sorbonne Université, INSERM U1138, Centre de Recherche des Cordeliers, Paris, France; 14 Departement de Biologie et Pathologie Médicales, Gustave Roussy Cancer Campus, Villejuif, France; 15 CNRS UMR2001, Paris, France; 16 Departement de Médicine Oncologique, Gustave Roussy Cancer Campus, Villejuif, France; 17 Cancer Research Institute Ghent (CRIG), Ghent, Belgium; 18 Cell Biology and Metabolomics Platforms, Gustave Roussy Cancer Campus, Villejuif, France; 19 Pôle de Biologie, Hôpital Européen Georges Pompidou, Assistance Publique-Hôpitaux de, Paris, France; 20 Department of Women’s and Children’s Health, Karolinska University Hospital, Stockholm, Sweden; 21 Suzhou Institute for Systems Medicine, Chinese Academy of Medical Sciences, Suzhou, China; 22 Center of Clinical Investigations in Biotherapies of Cancer (CICBT) 1428, Villejuif, France; 23 Present Address: Clinical Cooperation Unit Applied Tumor Immunity, National Center for Tumor Diseases (NCT), German Cancer Research Center (DKFZ), Heidelberg, Germany

**Keywords:** Tumour biomarkers, Tumour biomarkers

## Abstract

Ileal epithelial cell apoptosis and the local microbiota modulate the effects of oxaliplatin against proximal colon cancer by modulating tumor immunosurveillance. Here, we identified an ileal immune profile associated with the prognosis of colon cancer and responses to chemotherapy. The whole immune ileal transcriptome was upregulated in poor-prognosis patients with proximal colon cancer, while the colonic immunity of healthy and neoplastic areas was downregulated (except for the Th17 fingerprint) in such patients. Similar observations were made across experimental models of implanted and spontaneous murine colon cancer, showing a relationship between carcinogenesis and ileal inflammation. Conversely, oxaliplatin-based chemotherapy could restore a favorable, attenuated ileal immune fingerprint in responders. These results suggest that chemotherapy inversely shapes the immune profile of the ileum–tumor axis, influencing clinical outcome.

## Introduction

The field of cancer treatment has seen major advancements over the last few years with the approval of immune checkpoint inhibitors (ICI) for multiple indications. However, ICIs do not benefit all patients; there are still major challenges to overcome primary or secondary resistance against immunotherapy. For colorectal cancer (CRC), microsatellite stability (MSS) is resistant to ICI and represents the vast majority of CRC [1]. Combination chemotherapy based on the use of 5-fluorouracil (5-FU) plus oxaliplatin (OXA) (FOLFOX) is routinely used as first-line treatment for advanced CRC [2], although with no curative intention. Therefore, it is important to improve treatment efficacy by combining standard chemotherapy with ICI. To do so, the identification of new therapeutic options and associated biomarkers will help selecting patients for clinical trials and evaluating therapeutic responses.

Recently, the interaction between the gut-associated immune system (GALT) and the immune tumor microenvironment (TME) has been recognized. Evidence from preclinical models and patients unraveled the gut microbiota and local immunity as a crucial crossroad in the modulation of responses to cancer therapies (reviewed in ref. [3]). The gut immune response is the result of a fine-tuned homeostatic equilibrium between immunity to pathogenic antigens and tolerance to nonpathogenic ones. Interactions between the microbiota, the intestinal epithelial barrier, and GALT continuously shape the function of the gut immune system [4]. Chemotherapy can affect the integrity of the gut epithelial barrier, thus altering the outcome of the local and systemic immune response.

Our group provided mechanistic insights into how the microbiota and apoptosis of intestinal epithelial cell (IEC) in the ileum elicit an immune response against proximal colon cancer (pCC) in the context of OXA-based chemotherapy [5]. The local priming of immune cells in the mesenteric lymph nodes (mLN) culminated in the enrichment of CD3^+^ T cells, CD8^+^ cytotoxic T lymphocytes (CTL), and T follicular helper (Tfh) in the TME, indicating a favorable immune contexture [6, 7] associated with good prognosis and treatment response [8]. Whether and how the local mucosal immune profile was impacted remained unexplored. In the present study, we describe ileal mucosal immune changes in pCC patients and their association to clinical outcome. Furthermore, we demonstrate cause–effect relationships among the different factors involved in shaping ileal immunity using relevant experimental mouse models of colon cancer.

We reveal that favorable tumor immunity and clinical outcome are associated with a relative loss of immune markers in the ileum. Using spontaneous and transplantable colon cancer mouse models, we identified functional links among tumor, intestinal microbes, and chemotherapy impacting on ileal immunity, resulting in apparently opposite changes between ileal inflammation and tumor immunosurveillance. We believe that these findings are important for the future development of new treatment combinations against colon cancers.

## Results

### Ileal immune parameters dictate pCC patients’ prognosis

We recently provided insights into how ileal IEC death and the local microbiota elicit a systemic immune response against pCC during OXA-based chemotherapy [5]. Indeed, we showed that Tfh primed in the mLNs post OXA accumulates in colonic tumor beds instead of being redirected toward the chemotherapy-inflamed ileum. In these pCC patients, we analyzed the ileal immune tonus and its impact on patient survival.

Right hemicolectomy is the standard surgical treatment for malignant neoplasms of the right colon, which involves the resection of the tumor along with nonmalignant tissues, including the terminal ileum. This procedure allowed us to concomitantly monitor the ileal and tumoral immune parameters, the ileal and colonic microbiota lining healthy tissues, and pCC patient prognosis.

**Fig. 1:**
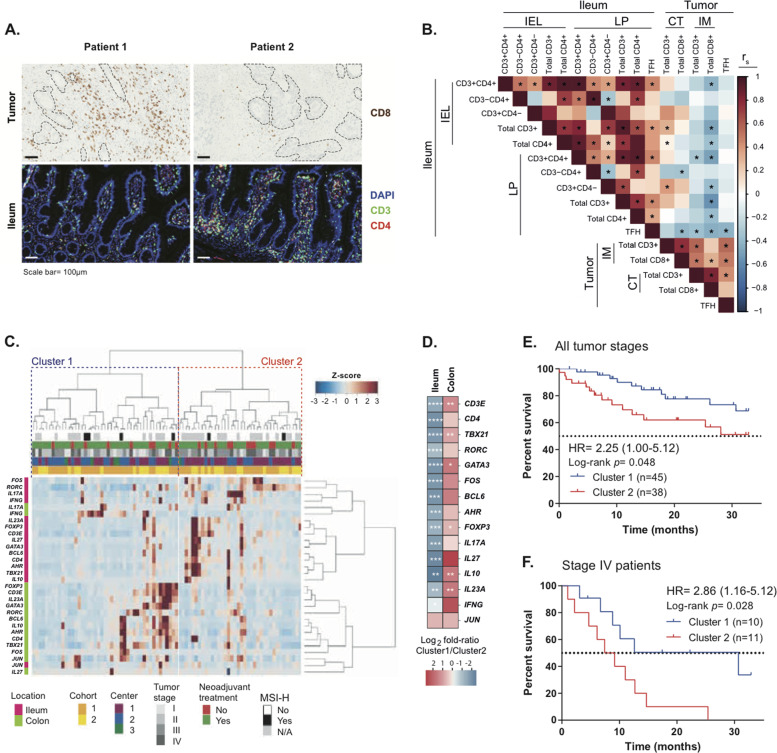
Ileal immune parameters dictate pCC patients’ prognosis. **A** Representative micrographs of (top panel) CD8^+^ T-cell infiltrates in the tumor core by immunohistochemistry (IHC) with delineation of tumor areas by dotted black lines and (bottom panel) CD3^+^ and CD4^+^ T-cell infiltrates by immunofluorescence (IF) staining in autologous ilea of two distinct pCC patients. Scale bar = 100 µm. **B** Heatmap showing Spearman correlation coefficients between autologous tumor and ileum immune cell densities in 53 distinct pCC patients for whom paired samples were available. *P < 0.05. IEL intra-epithelial lymphocytes, LP lamina propria, CT core of the tumor, IM tumor invasive margin. **C** Dendrogram and heatmap depicting the agglomerative hierarchical clustering of pCC patients (*n* = 83, columns) according to ileal and colonic immune gene transcription (rows) at surgery. Distance was measured with 1–Pearson correlation coefficient and agglomeration with Ward’s method. Clinical variables and tissue of origin are indicted by the corresponding color code on the top and side border, respectively. **D** Heatmap of gene expression patterns in autologous ileum and colon as a log_2_ fold ratio (FR) between cluster 1 and cluster 2 individuals. Mann–Whitney *U* test: *P < 0.05; **P < 0.01; ***P < 0.001; ****P < 0.0001. **E**, **F** Kaplan–Meier curves for time to treatment failure (progression or cancer-related death) segregated according to the clustering from (**C**) analyzed by Mantel–Cox regression test in 83 pCC patients (**E**) or only in stage IV metastatic pCC patients (**F**).

Immunofluorescence-based staining of T-cell subsets in the ilea and tumor beds revealed an inverse correlation between CD3^+^ and CD4^+^ T lymphocytes within the ileal epithelium (EP) or lamina propria (LP), and CD8^+^ tumor-infiltrating lymphocytes (TILs) in the invasive margins of the resected pCC (Fig. 1A, B). Hence, cancer T-cell infiltration (a surrogate marker of prognosis in CRC) [9] is particularly dense in patients whose ilea contain few T cells. Notably, ileal LP Tfh cells exhibited robust anticorrelations with TILs, including tumor-infiltrating Tfh cells (Fig. 1B). We next evaluated T lymphocyte-related gene expression patterns, focusing on a custom panel comprising 15 transcription factors and cytokine genes, by means of quantitative real-time polymerase chain reaction (RT-qPCR) performed on the terminal ileum and proximal colon segments removed during surgery. This RT-qPCR reaction was validated by protein expression for selected gene products, such as for the aryl hydrocarbon receptor (AHR) for which gene and protein expressions markedly correlated among each other (Supplementary Fig. S1A, B). Nonsupervised hierarchical clustering of immune gene products yielded two opposite clusters of patient samples, based on the geodistribution of gene expression, i.e., cluster 1 in which the mRNAs were scarcely expressed in the ileum but highly expressed in the colon, and cluster 2 in which mRNAs exhibited an opposite pattern with high expression in the ileum and low expression in the colon (Fig. 1C, D). Indeed, *CD3E*, *TBX21*, *FOXP3*, *IL10*, *GATA3*, and *IL23A* were more expressed in the colon while being less expressed in the ileum in cluster 1 compared with cluster 2 (Supplementary Fig. S2, right and left panels). Importantly, these gene expression profiles had a clinical relevance in thus far that cluster 1 patients exhibited a prolonged time to treatment failure (progression or cancer-related death) than those individuals whose ileal signature fell into cluster 2 (Fig. 1E, clinical characteristics of the patients are detailed in Table S1, adjusted *P* values in Table S2). Importantly, ileal immune gene expression was independent of the disease stage, and ileal immune tonus even predicted survival of stage IV pCC metastatic patients (Fig. 1F).

Altogether, we conclude that the immune tonus of the ileal mucosa correlates with colon cancer immunosurveillance, as well as with patient survival.

### Colon cancer promotes high ileal immune gene expression

**Fig. 2:**
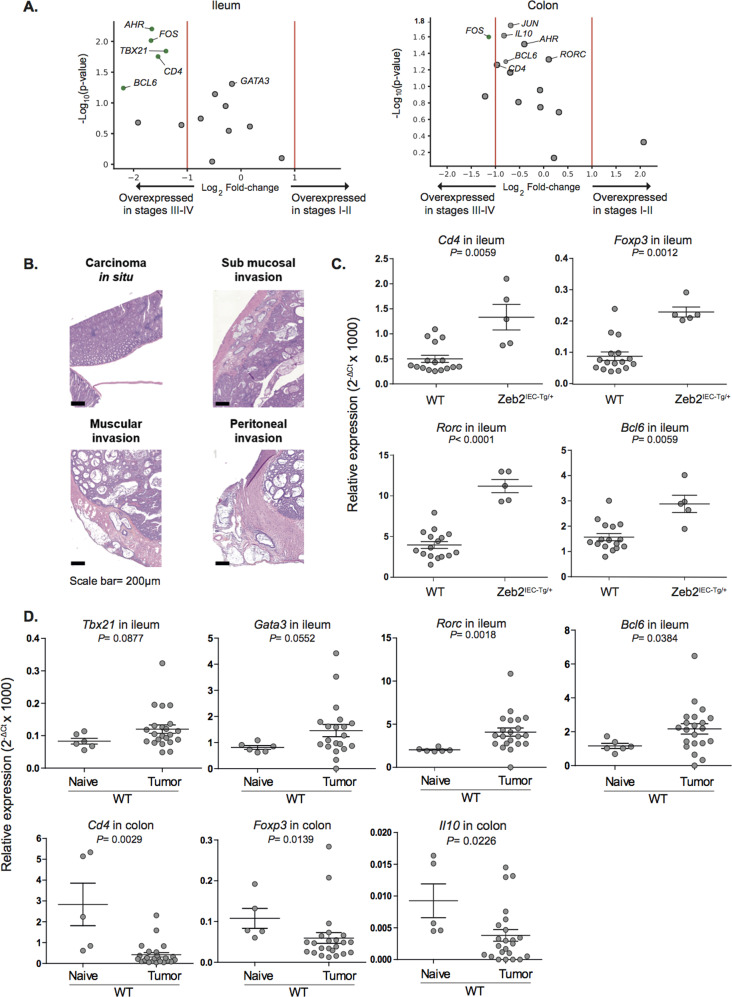
Inverse relationship between ileal and cancer immune tonus in CC models. **A** Volcano plots of the differential immune gene transcription in qPCR of ileum (**A**) and colon (**B**) mucosae contrasting stage I–II versus stage III–IV pCC patients (TNM staging system). Volcano plots were generated computing for each gene product expressed in intestinal mucosae of 83 pCC patients: (i) the log_2_ of fold change (FC) among the mean relative abundances of transcripts after normalization in early versus advanced disease stages (*x* axis); (ii) the co-log_10_ of *P* values deriving from Mann–Whitney *U* test calculated on relative abundances in absolute values (*y* axis). Green and gray dots are considered significant (*P* < 0.062) or not (*P* > 0.062), respectively. **B** Representative micrographs of hematoxylin and eosin (H&E)- stained sections of intestinal samples collected from *Zeb2*^*IEC-Tg/+*^ mice showing different tumor progression. Scale bar: 50 µm. **C** Relative expression of immune genes in ilea of *Zeb2*^*IEC-Tg/+*^ mice and WT littermates as assessed by RT-qPCR. One dot represents one ileum and the pooled data of all ilea are shown. Mean ± SEM are depicted. Mann–Whitney *U* test *P* values are shown. **D** Relative expression of immune genes in ilea and colon of MC38-bearing mice WT versus naive WT mice as assessed by RT-qPCR. One dot represents one ileum or one colon and the pooled data of all ilea or colons are shown. Mean ± SEM are depicted. Mann–Whitney *U* test *P* values are shown.

Although the ileal immune tonus was independent of tumor stage (Fig. 1C, F), genes coding for *CD4*, the Tfh-relevant transcription factor *BCL6*, the T helper 1-relevant transcription factor *TBX21*, and *AHR* were significantly (>2-fold) increased in the ileum (Fig. 2A, left panel) but not in the colon (Fig. 2A, right panel) in more advanced pCC stages (III/IV) compared with early stages of colon carcinogenesis.

Intrigued by these findings, we turned to mouse models to validate the observation that tumor progression could indirectly stimulate ileal inflammation/immunity. IEC-specific transgenic expression of the epithelial–mesenchymal transition regulator Zeb2 in mice (*Zeb2*^*IEC-Tg/+*^ mice) leads to increased intestinal permeability and spontaneous invasive colon carcinoma development in a microbiota-dependent manner [10]. At a young age, *Zeb2*^*IEC-Tg/+*^ mice develop dysplasia of the colon EP that progresses to neoplastic lesions [10], but maintain a normal small intestinal EP. Histological examination of colon tissues revealed different grades of tumor progression in individual mice. Among mucinous tumors in the proximal region of the colon, we observed cancers with clear signs of transmural invasion and growth throughout the muscularis mucosae until peritoneum invasion (Fig. 2B). The presence of invasive tumors in *Zeb2*^*IEC-Tg/+*^ mice was associated with increased expression of ileal immune genes (such as *Cd3e, Cd4, Ahr, Rorc, Bcl6, Gata3, Il10, Il23a,* and *Il27*) compared to *Zeb2*^*IEC–+/+*^ wild-type (WT) littermates (Fig. 2C, Supplementary Fig. S3A and Table S3). RT-qPCR examination of these tumor areas revealed a positive correlation between tumor burden and several immune genes found in ilea from pCC patients, such as *Cd4, Bcl6*, and *Gata3* (Supplementary Fig. S3B).

We next investigated whether shifts in the gut microbiota might explain these ileal alterations during the colon carcinogenesis program. Of note, rederivation of *Zeb2*^*IEC-Tg/+*^ mice in germ-free (GF) conditions completely abrogated cancer development [10]. Mice reared in specific pathogen-free (SPF) conditions exhibited a more pronounced Th1/Treg profile and an attenuated Il-17 tonus in their ilea as compared to axenic *Zeb2*^*IEC-Tg/+*^ (Supplementary Fig. S3C). However, during colon carcinogenesis, ilea from SPF *Zeb2*^*IEC-Tg/+*^ still manifested a relative overexpression in *Il10, Gata3*, and *Il27* genes (Supplementary Fig. S3C and Table S4), suggesting that this ileal immunomodulation did not depend on the ileal microbiome.

To validate the notion that cancer can induce ileal inflammation, we switched to a transplantable colon tumor model that can be inoculated subcutaneously (s.c.) into syngeneic immunocompetent mice. We compared the ileal immune transcriptome of mice bearing s.c. MC38 cancers with that of naive tumor-free mice. Here again, we confirmed that the ileal mRNAs encoding *Gata3*, *Bcl6*, *Rorc*, and *Tbx21* were increased in tumor bearers at day 10 of implantation compared to naive counterparts. In contrast, *Cd4*, *Foxp3*, and *Il10* transcription were reduced in the colon (Fig. 2D).

These results indicate that the presence of colon carcinogenesis is accompanied by a higher ileal immune tonus, paralleling tumor progression in human and mice.

### Chemotherapy alters the cancer-induced ileal immune activation

The GALT provides specific types of immune responses to local challenges along the gastrointestinal tract. We recently showed that the ileal crypt cells were the main targets of OXA-based chemotherapy with respect to apoptosis induction [5]. Driven by this observation, we evaluated the impact of systemic cytotoxicants on the ileal immune signature.

**Fig. 3:**
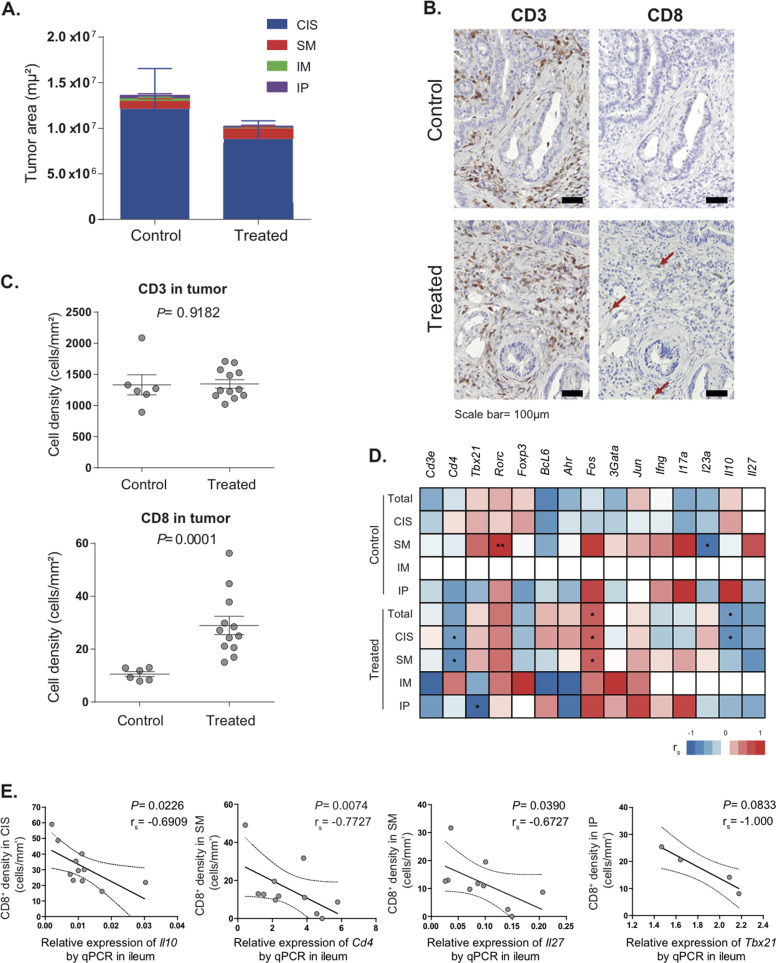
Ileal immune parameters are restored by OXA-based chemotherapy. **A** Assessment of tumor loads by quantification of tumor areas in in situ, submucosal, muscular, and peritoneal tumors in *Zeb2*^*IEC-Tg/+*^ mice treated with 5-FU + OXA or vehicle (PBS control). **B** Representative micrograph of CD3^+^ (left panel) and CD8^+^ (right panel, indicated with arrows) cell infiltrates by IHC staining in the tumors of *Zeb2*^*IEC-Tg/+*^ mice treated by 5-FU + OXA or PBS control. Scale bar: 50 µm. **C** Quantification of CD3^+^ (upper panel) and CD8^+^ (lower panel) cell densities in tumors of *Zeb2*^*IEC-Tg/+*^ mice (relative to IHC staining in B). Mann–Whitney *U* test *P* values are shown. **D** Heatmap showing Spearman correlation coefficients between ileum immune gene expression and CD8^+^ cell densities in tumors (quantified in C) of *Zeb2*^*IEC-Tg/+*^ treated by 5-FU + OXA (or PBS control). CD8^+^ TILs were assessed by IHC and quantified in tumor areas with different degree of invasion. Significant correlations are indicated, *P < 0.05. CIS carcinoma in situ, SM submucosal tumor, IM muscle-invasive tumor, IP invasive tumor in the peritoneum; Total: total tumor area. **E** Spearman correlation between ileum immune genes and CD8^+^ cell densities in tumor. CD8^+^ TILs were assessed by IHC and quantified in tumor areas with different degrees of invasion. One dot represents one mouse; the black and dotted lines show the regression line and 95% confidence intervals, respectively. Spearman coefficients and *P* values are shown in the figures. CIS carcinoma in situ, SM submucosal tumor, IP invasive tumor in the peritoneum.

Given that FOLFOX is the first-line standard chemotherapy for most patients with advanced CRC [2], we adapted this treatment to the mouse model, treating *Zeb2*^*IEC-Tg/+*^ mice with a mixture of 5-FU and OXA. Histological examination of the colons and quantification of tumor invasion (as in Fig. 2B) revealed a mild response to therapy compared to PBS-treated controls (Fig. 3A), as expected given the aggressiveness of this model [10]. However, we did observe a chemotherapy-induced immune response in the TME with a significant increase in CD8^+^ cells, though no alteration in the frequency of CD3^+^ T cells (Fig. 3B, C). We found a negative correlation between the expression of ileal immune genes and the frequency of CD8^+^ TILs only in chemotherapy-treated mice (Fig. 3D), suggesting that chemotherapy partially dampened the ileal immune signature (*Cd4*, *Tbx21*, *Il10,* and *Il27*) associated with carcinogenesis (Fig. 3E).

In conclusion, tumor infiltration showed a negative correlation with ileal immune tonus after chemotherapy in an aggressive spontaneous mouse model of colonic carcinogenesis.

### Bacteria regulate the antitumor efficacy of OXA by shaping the ileal immune profile

**Fig. 4:**
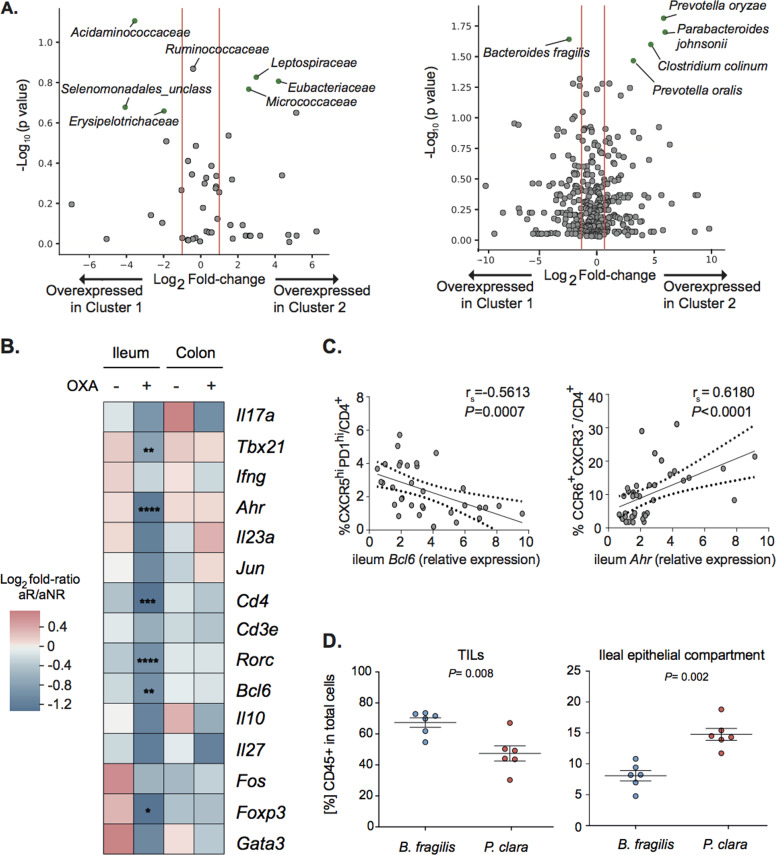
Microbiome composition regulates the antitumor efficacy of OXA by shaping ileal immune profile in experimental models. **A** Volcano plot representation of differential microbiota composition at family (**A**) and species (**B**) levels matching cluster 1 versus cluster 2 pCC patients as defined in Fig. 1C. Volcano plots were generated computing for each bacterial family (left) or species (right) residing in ileal mucosae of 83 pCC patients: (i) the log_2_ of FR among the mean relative abundances in cluster 1 versus cluster 2 (*x* axis); (ii) the co-log_10_ of *P* values deriving from Mann–Whitney U test calculated on relative abundances (*y* axis). Green dots are considered significant at *P* < 0.05 (gray dots *p* > 0.05). B. Heatmap of the Log2 FR of the immune gene transcript levels between aNR and aR wild-type mice in PBS and OXA-treated groups in ilea and colons at sacrifice (day 21). Mann–Whitney *U* test: *P < 0.05; **P < 0.01; ***P < 0.001; ****P < 0.0001. **C** Spearman correlations between ileal *Bcl6* gene transcripts and percentages of Tfh (left panel) and between ileal *Ahr* gene transcripts and percentages of CCR6^+^CXCR3^–^/CD4^+^ T cells determined by FACS in tdLN in OXA-treated avatar groups at sacrifice (day 21 after treatment). The black and dotted lines show the regression line and 95% of confidence intervals, respectively. Spearman coefficients and *P* values are shown in the figures. **D** Flow cytometry analysis of CD45^+^ lymphoid cell infiltration in tumor and ileal epithelial compartment of MC38 tumor-bearing mice treated with OXA and gavaged with *B. fragilis* or *P. clara*. Each dot represents one tumor or one ileum at sacrifice (day 4 after treatment). Means ± SEM are depicted. Mann–Whitney *U* test *P* values are shown.

Ileal apoptosis may affect the composition of the ileal microbiota and therefore influence immune responses in the GALT and tumor beds [5]. Therefore, we evaluated the fecal and ileal microbiota of our cohort of pCC patients [5]. An abnormal (or “dysbiotic”) fecal microbial repertoire has been detected during adenoma–carcinoma transformation [10] as well as in CRC patients [11–13]. We performed 16 S rRNA gene sequencing of gene amplicons of ileal mucus from our previously described [5] pCC cohort (*n* = 70). Volcano plots revealed an overabundance of *Prevotellaceae* family members in ileal samples from patients diagnosed at advanced pCC stages (i.e., *Prevotella buccalis* and *Prevotella denticola*) compared with earlier stages (Supplementary Fig. S4). We next examined the ileal immune signature in relation to the ileal microbiome assessed by 16S rRNA gene sequencing of the same patients. Surprisingly, the ileal microbiota could segregate patients classified according to their ileal immune gene expression. At the level of bacterial families or classes, Volcano plots highlighted an overabundance of the *Erysipelotrichaceae* family and the *Negativicutes* class (such as *Acidaminococcaceae*, *Selenomonadales unclass*.) in cluster 1 patients (with better prognosis than cluster 2 patients) (Fig. 4A, left panel). At the species level, there was a significant enrichment in *Prevotella* spp. (*P. oralis, P. oryzae*) in cluster 2 patients with dismal prognosis compared to cluster 1 (Fig. 4A, right panel). Conversely, the only bacterium enriched in the favorable cluster 1 (compared with cluster 2) was *Bacteroides fragilis* (Fig. 4A, right panel), in line with the previously reported association of this species with an immunogenic ileal microbiota [5].

To establish a cause–effect relationship between the microbiota composition and the ileal prognostic signature, we took advantage of the “avatar” model in which GF mice (previously shown to be nonresponders to OXA [14]) received fecal microbiota transplantation (FMT) from human pCC patients and hence were “humanized” with respect to a potentially pathogenic intestinal microbiota [5]. In these gut experiments of “humanization”, some patient feces (8/12 cases) yielded an avatar responder (aR) phenotype in the mice, meaning that subsequently implanted MC38 responded to OXA treatment, while others yielded an avatar nonresponder phenotype (aNR) [5]. OXA-treated aR (but not aNR) mice showed a depletion of the mRNAs coding for *Cd4, Tbx21, Rorc, Ahr, Bcl6*, and *Foxp3* from their ilea (Fig. 4B). This difference between aR *versus* aNR transcriptomes was only found in the ileum, not in the colon (Fig. 4B and Supplementary Table S5).

Of note, we previously reported that aR mice exhibited a stronger increase in Tfh cells in tumor- draining lymph nodes (tdLNs) than aNR mice [5]. The abundancy of Tfh in tdLN modulated by FMT negatively correlated with the expression of *Bcl6* in the ileal mucosa (Fig. 4C, left panel), confirming the negative relationship between ileal and tumor-specific immunity outlined in Fig. 1 in patients. Conversely, tdLNs from aNR mice contained higher CCR6^+^CXCR3^–^ CD4^+^ Th17 cells (known to be associated with an immuno-suppressive milieu [15]) than those from aR mice, and such cells exhibited a positive correlation with ileal *Ahr* mRNA levels, AhR being a transcription factor associated with Th17 differentiation [16] (Fig. 4C, right panel).

Ileal immune responses mainly take place in mLNs where T cells acquire a gut tropism and relocalize within intestinal EP or LP [17]. However, lymphocytes can dynamically migrate through the lymphatic space to find a cognate antigen in other locations, including extraintestinal tumor sites by various homing mechanisms [18]. To test the impact of the microbiota in altering migration patterns of lymphocytes primed in the gut, we performed oral gavages with bacteria associated with favorable (such as *Bacteroides fragilis*) or dismal prognosis (such as *Paraprevotella clara)*. We administered this oral supplementation twice, 24 hours before and after OXA i.p. injection. Here again, we found a mirror image of the leukocyte infiltration induced by orally administered bacteria in the ileal mucosa and the tumor bed. *B. fragilis* was associated with higher TILs and lower CD45^+^ cells in the ileal compartment, compared to *P. clara*-treated groups (Fig. 4D).

The aforementioned results support the notion that the ileal microbiota shapes local immune responses, which then control colon cancer immunosurveillance.

## Discussion

Here, we demonstrate that the prognosis of pCC patients is associated with an ileal immune fingerprint that inversely correlates with the colonic signature and appears to be linked to tumor immunosurveillance.

First, we highlighted in a cohort of 83 pCC patients that those individuals presenting with a low ileal immune gene transcriptome have a favorable prognosis, even when diagnosed with a stage IV pCC (Fig. 1C, F). Second, the CD3^+^CD4^+^ T cells of IELs and LP inversely correlated with CD3^+^CD8^+^ counts, a central parameter of the immunoscore [7] (Fig. 1B). Indeed, scarce expression of immune genes in the ileum corresponded to high immune gene expression in healthy colons, specifically for gene products of the Th1 (*CD4*, *TBX21*), Th2 (*GATA3*), and Treg (*FOXP3*, *IL10*) differentiation profile, while the Th17 pattern (centered around *IL17A*, *AHR*, and *RORC*) was relatively stable across the small and large parts of the intestine (Supplementary Fig. 2). This might account for the colon cancer infiltration of those Th1/Th2 cells in the core of the tumor in favorable cases.

Conversely, the ileal immune tonus was affected by colonic carcinogenesis as indicated by the fact that the since growth of heterotopic (s.c. MC38) or orthotopic (EMT-induced Zeb2-transgenic) colon cancers induced this upregulation of ileal immune gene products (Fig. 2C, D). At this point, it remains an open question how tumors can send proinflammatory signals to the ileum.

We also found evidence that OXA-based chemotherapy reshuffles the ileal immune system, favoring the reduction of immune transcription of a variety of gene products in MC38-bearing avatar mice as well as in Zeb2-transgenic animals (Figs. 3 and 4). The striking negative correlations found in the *Zeb2*^*IEC-Tg/+*^ and s.c. MC38-transplantable models between the ileal and tumor immunity post-OXA-based therapy suggest that antigen-specific T cells could be primed in mLNs and migrate back to tumor beds instead of returning to the inflamed ileal mucosa. Although speculative, this is in line with our previous findings obtained in s.c. MC38 in which OXA generated an immunogenic cell death of ileal IEC that contributed to elicit Tfh in mLNs and tdLNs and fosters CD8^+^ T-cell-dependent tumor control [5]. We reported that OXA treatment triggered the migration of ileal CD11b^–^CD103^+^ LP-DCs to the mLN and their upregulation of IL-12 and IL-1β in a gut microbiota-dependent manner [5]. We also reported that migratory LP-DCs from the terminal ileum contributed to Tfh priming post OXA [5]. It is tempting to speculate that T cells primed in mLN may either traffic back to the ileal inflammation caused by OXA-induced apoptosis of the crypts or instead are redirected to tumor lesions. It is conceivable that the chemokine axis is reshuffled by cytotoxic agents in both ileal and tumor epithelia in an inversed manner, changing the homing equilibrium between gut to tumor beds, as previously shown for CCR9^+^ T cells [19, 20].

Functional links between ER stress in the epithelial barrier and immune activation in the TME were recently reported in *Rnf5*^*−/−*^ mice [21]. RNF5 is a membrane-anchored E3 ubiquitin ligase implicated in endoplasmic reticulum (ER)-associated protein degradation. Decreased secretion of antimicrobial peptides and increased cell death in the ileal crypts caused intestinal dysbiosis in *Rnf5*^*−/−*^ mice. This bowel injury allowed the recruitment, activation, and mobilization of CCR7-expressing dendritic cells to Peyer patches, mLN [22], and tdLN, culminating in the massive infiltration of IFNγ-producing CD4^+^ and CD8^+^ TILs and tumor control of poorly immunogenic melanoma [21]. The microbiota shift in *Rnf5*^*−/−*^ mice was underscored as a key factor in the immunosurveillance mechanisms. This report is in line with our findings showing that some intestinal commensals can affect the ileal immune system during OXA-induced cancer immunosurveillance. By segregating responders from nonresponders in “humanized” mice bearing colon cancers, we demonstrated that the OXA-induced reduction of tumor size was associated with low ileal immune transcription (Fig. 4B, C), paralleling Tfh accumulation and Th17 reduction in tdLNs in responders. Patients diagnosed with pCC belonging to cluster 1 (displaying a low ileal immune profile, Fig. 1C, D) presented a relative dominance of *B. fragilis* and an underrepresentation of *Prevotella* spp., these two species creating an opposite balance of the leukocyte infiltrates in the ileum and tumors (Fig. 4D). In our previous work [5], we showed that *B. fragilis* contributes to IL-12- and IL-1β-dependent priming of Tfh in tdLNs. Other reports proposed alternative mechanisms on how microbial species could modulate T-cell patrolling patterns during carcinogenesis. Microbial species may induce a burst of chemokines in GALT or in the TME, thus affecting the trafficking of helper and effector T cells [21]. In CRC, the commensals may act on cancer cells to modulate their capacity to produce chemokines that may govern the immunoscore and patient prognosis through the recruitment of Th1, Tfh, or Th17 cells into the tumor bed [23]. In hepatocellular carcinoma, the gut microbiome uses bile acids as messengers to regulate chemokine CXCL16 levels on liver sinusoidal endothelial cells in order to control the accumulation of CXCR6^+^ hepatic natural killer T (NKT) cells that keep liver tumors at bay [24]. In contrast, lipoteichoic acid, a cell wall component of Gram-positive bacteria, enhances the senescence-associated secretory phenotype of hepatic stellate cells in collaboration with a gut microbial metabolite, deoxycholic acid, and upregulates the inflammatory cascade and COX2 in a TLR2-dependent manner. In turn, COX2-mediated prostaglandin E2 production suppresses the antitumor immunity, thereby contributing to hepatocellular carcinoma progression [25].

Previous reports using oral administration of anticancer probiotics analyzed the immunomodulatory effects of these live biotherapeutics on the colonic mucosae, highlighting the accumulation of IFNγ-producing CD8^+^ T cells as a surrogate marker of tumor immunosurveillance of cancers established in gut or extraintestinal locations. Tanoue et al. used a 11-strain-based product that elicited potent cytotoxic T-cell responses, including tissue-resident T cells devoid of colitis-inducing properties that were associated with ICI-induced anticancer effects against melanoma [26]. Wang et al. administered *Akkermansia muciniphila* and its bacterial TLR4 derivative A-muc_1100 that both prevented colitis-induced CRC and CT26 by inducing the colonic accumulation of TNFα-producing CD8^+^ T cells elicited in mLN and spleens [27]. In these reports, including ours, it is unclear to which extent these CTL responses elicited from the gut and redirected to TME contribute to the antitumor effects. Some of these CTL responses might be specific for bacterial antigens, sharing some molecular mimicry with tumor epitopes [28, 29] or be directly presented by MHC class I molecules on tumor cells that were primarily infected by such bacteria [30, 31]. More work is needed at the TCR and single-cell level to elucidate the relevance of such TILs for cancer immunosurveillance.

**Fig. 5:**
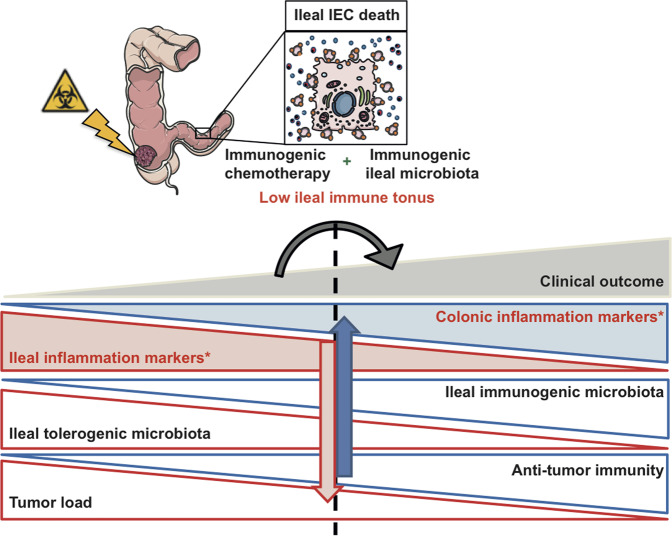
The interactive dialog between the microbiota, the immune system, and the epithelial barrier in the ileal mucosae of colon cancer patients. Ileal IEC apoptosis together with natural adjuvants from distinct commensals elicit an immune response against self-antigens (likely derived from crypt/villus-progenitor cells) that eventually infiltrates proximal colon cancers (pCC) as demonstrated in Roberti et al. [5]. Here we show the relevance of ileal mucosal immune transcriptome on the prognosis of pCC patients and its connection with ileal microbiota. In advanced staging or nonresponders to adjuvant therapies, the whole immune ileal transcriptome remains upregulated, while immune gene products encoding transcription factors and cytokines are downregulated in the healthy colon mucosae. This mirror image between ileum and colon of the inflammation and immunity fingerprints in the lamina propria and/or epithelial areas may reflect a coordination of the two compartments to face the challenge of tumor progression. The empty boxes refer to our previous work reported in Roberti et al. [5]. The colored boxes and arrows with stars* represent the follow-up study proposed in this paper.

Regardless of the detailed mechanism defining the ileal signature, some clinically relevant issues may arise from our data. First, based on our previous [5] and the present multidimensional analyses (including microbial composition, and immune-related gene expression profiles), we surmise that the ileal mucosa represents the intestinal compartment that most closely determines the prognosis of advanced pCC patients (Fig. 5). Given that sidedness has recently been shown as a major negative prognostic factor in metastatic CRC [32–34], stratification of stage IV pCCs based on the ileal immune signature has great potential for decision-making, regarding adjuvant therapies. A high preexisting cytotoxic T-cell infiltrate in the TME predisposes to sensitivity to ICI [35, 36]. While most biomarkers relate to the immune contexture of the primary CRC [9], the present data offer alternative predictors of response to OXA and its combination with ICI by assessing the ileal fingerprint (immune gene expression pattern and microbiome composition) to address the prognosis of metastatic patients. This signature can be routinely studied upon pCC surgery, taking advantage of the systematic resection of the terminal ileum. Further prospective work is ongoing in our laboratories to validate these novel biomarkers in MSI and MSS CRC. In addition, our present and previous [5] findings strongly advocate for the use of ileum and crypt-targeting cell death inducers in combination with such immunotherapies to convert “cold” into “hot” pCCs, regardless of their MSI status.

## Materials and methods

### Patients

In a cohort of pCC patients who underwent hemicolectomy, we previously reported the epithelial and microbial parameters that were significantly associated with outcome [5]. The inclusion criteria for the study were patients with colon adenocarcinoma who underwent right hemicolectomy plus ileal resection (last 10 centimeters). The number of samples was selected based on availability of the samples. Patients were not on antibiotics at the moment of surgery. Four different health cancer centers participated in the study: Gustave Roussy Cancer Campus (Villejuif, France), Centre G-F Leclerc (Dijon, France), Hôpital Bicêtre (Le Kremlin-Bicêtre, France), and Centre hospitalier universitaire-Estaing (Clermont Ferrand, France). Clinical studies were conducted according to the ethical guidelines and approval of the local CCPPRB. The study received the legal authorization by the Ministère de l’Enseignement Supérieur et de la Recherche (AC-2013-1884). Written informed consent in accordance with the Declaration of Helsinki was obtained from all patients.

### Patients’ clinical categorization and follow-up

The information pertaining to tumor staging (TNM staging system of the American Joint Committee on Cancer—AJCC) and MSI status was obtained in pathology reports. Information pertaining to cancer recurrence, death, and causes of death was obtained from hospital records. All observations were censored at loss to follow-up and at the end of the study period. OS was measured from the date of surgery to the date of death from any cause. Time to treatment failure (TTF) was measured from the date of surgery to the date of local or regional recurrence, distant metastases, or death from cancer and treatment-related death; patients were censored at non-cancer-related death (TTF study period 1000 days).

### Preparation of patients’ samples for the study

Samples were collected at the time of surgery. Fecal content from colon for FMT experiments and mucus adherent to the ileum mucosae for 16 S rRNA gene sequencing were already described [5]. *Mucosal biopsies*: approximately 4 cm^2^ of ileal and nontumoral colonic tissue (distant from the tumor) that included at least the submucosa was removed, cut into small pieces (approximately 1 cm^2^), and transferred in sterile cryotubes. All samples were immediately frozen at −80 °C after collection. Tumor samples were retrieved from formalin-fixed paraffin-embedded (FFPE) tissue from the pathology archives. Paired analyses were done with the maximal number of available samples; there were no exclusions, except for samples that did not meet the quality control criteria. Patients’ clinical characteristics are detailed in Table S1.

### Mice

Female C57BL/6 mice were purchased from Harlan. Mice were used between 7 and 14 weeks of age. GF C57BL/6 were obtained from the facility located at CDTA (Cryopreservation, Distribution, Typage et Archivage, Orléans, France). *Zeb2*^*IEC-Tg/+*^ mice were previously described [10].

Axenic mice were housed in positive-pressure isolators. All experiments were performed on mice of C57BL/6 genetic background. Mice were housed in individually ventilated cages in a SPF animal facility. Experiments were performed at the animal facility in Gustave Roussy Cancer Campus, at the VIB Center for Inflammation Research and the GF facility of Ghent University. All experiments on mice were performed in compliance with institutional and European laws and regulations (Ministère de la Recherche, de l’Enseignement Supérieur et de l’Innovation, MESRI, and Ethical Committee for Animal Experimentation at Ghent University’s Faculty of Sciences and Faculty of Medicine and Health Sciences). Sample sizes were decided based on previous publications and experience and common standards in similar field, for calculating statistical significance. No animals were excluded from the analysis.

### Tumor immune profiling by immunohistochemistry in human samples

These results were previously reported [5]. In the present study, we reanalyzed them (*n* = 53) to perform correlations with the ileum immunity.

### Immunofluorescence staining, scanning, and analysis for CD3 and CD4 expression in ileum

For multiplexed staining, 3-µm-thick sections of formalin-fixed, paraffin-embedded ileal tissue were stained by automated immunostainer (DISCOVERY ULTRA, Ventana, IGR). Heat-induced antigen retrieval in Citrate buffer (pH 6.0) for 10 minutes at 100 °C was performed. Then, the slides were incubated on primary monoclonal rabbit anti-human CD4 antibody (Spring, SP35, 0.5 µg/mL) for 1 hour at 37 °C, detected by Discovery UltraMap anti-rabbit HRP (Ventana, #760-4315), and visualized by Discovery Cy5 kit (Ventana, #760-238). Heating step with Citrate Buffer was carried out, as described above. Next, the slides were incubated on primary polyclonal Rabbit anti-human CD3 antibody (DAKO, #A0452, 3 µg/mL) for 1 h at 37 °C, detected by Discovery UltraMap anti-rabbit HRP (Ventana, # 760-4315), and visualized by Discovery FAM kit (Ventana, # 760-243). After the heating step with Citrate Buffer, nuclei were subsequently visualized with Spectral DAPI (Perkin Elmer, FP1490, 1:10). Fluorescence analysis. Images displayed in the figures were acquired as whole-slide images (WSI) with a slide scanner Zeiss Axio Scan.Z1 (objective Plan-Apochromat 20×/0.8, 3CCD camera Hitachi HV-F202SCL) and exported from the Zeiss Zen 2 lite software as TIFF images. Some of the WSI were processed with using an algorithm developed in Visiopharm Integrator System (VIS) (Visiopharm A/S, Denmark). ROI was defined for each WSI by applying a threshold on the DAPI intensity, and then mean fluorescence intensity was measured in those ROIs.

### Characterization of gut immune gene expression profile by real-time quantitative PCR analysis

Total RNA from gut biopsies was extracted with RNeasy Mini Kit (Qiagen) and then reverse-transcribed into cDNA with the SuperScript III Reverse Transcriptase and the RNaseOUT™ Recombinant Ribonuclease Inhibitor (Life Technologies, Saint Aubin, France), in the presence of random primers (Promega, Charbonnieres, France) and the Deoxynucleoside Triphosphate Set, PCR grade (Roche Diagnostics, Meylan, France). cDNA was analyzed by real-time quantitative PCR (RT-qPCR) with the TaqMan method with TaqMan^®^ Gene Expression Assays using the Universal Master Mix II (Invitrogen) according to the manufacturer’s instructions using the 7500 Fast Real-Time PCR system (Applied Biosystems). Expression was normalized to the expression of the housekeeping gene of Beta 2 Microglobulin by means of the 2^−ΔCt^ method. All primers were from TaqMan^®^ Gene Expression Assay (Thermo Fisher).

Mouse primers: *B2m* (Mm00437762_m1), *Cd3e* (Mm01179194_m1), *Cd4* (Mm00442754_m1), *Tbx2*1 (Mm00450960_m1), *Ifng* (Mm01168134_m1), *Rorc* (Mm01261022_m1), *Il17a* (Mm00439618_m1), *Foxp3* (Mm00475162_m1), *Il10* (Mm01288386_m1), *Gata3* (Mm00484683_m1), *Il27* (Mm00461162_m1), *Il23a* (Mm00518984_m1), *Bcl6* (Mm00477633_m1), *Ahr* (Mm00478932_m1), *Fos* (Mm00487425_m1), and *Jun* (Mm00495062_s1).

For studies comparing naive mice to MC38-bearing WT mice: MC38 bearers bearing WT mice *n* = 21 and naive WT *n* = 6 mice have been tested.

For studies comparing WT mice (littermate controls) to *Zeb2*^*IEC-Tg/+*^ *n* = 16 samples from WT mice and *n* = 5 samples from *Zeb2*^*IEC-Tg/+*^ have been tested.

For studies comparing GF mice to SPF mice, WT SPF mice *n* = 16, WT GF mice *n* = 7, *Zeb2*^*IEC-Tg/+*^ SPF mice *n* = 5, and *Zeb2*^*IEC-Tg/+*^ GF mice *n* = 9 have been tested.

For studies characterizing gut immune gene expression profile in different tumor locations, nontreated *Zeb2*^*IEC-Tg/+*^ mice *n* = 6 and treated *Zeb2*^*IEC-Tg/+*^ mice *n* = 12 have been tested.

Human primers: *B2M* Forward: 5′-GATGAGTATGCCTGCCGTGT-3′; *B2M* Reverse 5′-AATTCATCCAATCCAAATGCG-3′; *B2M* Probe 5′-(6FAM) AACCATGTGACTTTGTCACAGCCCAA(TAM)-3′, *CD3E* (Hs01062241_m1), *CD4* (Hs01058407_m1), *TBX21* (Hs00894392_m1), *IFNG* (Hs00989291_m1), *RORC* (Hs01076112_m1), *IL17A* (Hs00174383_m1), *FOXP3* (Hs01085834_m1), *IL10* (Hs00961622_m1), *GATA3* (Hs00231122_m1), *IL27* (Hs00377366_m1), *IL23A* (Hs00372324_m1), *BCL6* (Hs00153368_m1), *AHR* (Hs00169233_m1), *FOS* (Hs04194186_s1), *JUN* (Hs01103582_s1). A total of *n* = 83 patients have been tested.

Analyses were done with the maximal number of available samples; there were no exclusions, except for samples that did not meet the quality control criteria.

### Chemotherapeutic treatment of Zeb2-transgenic mice

All chemotherapies were obtained from the Gustave Roussy Cancer Campus. Mice were allocated to groups ensuring an equal distribution of genders. When mice were 6 weeks old, 5-FU and OXA were administered i.p. at 50 mg/kg and 10 mg/kg, respectively. A second treatment with OXA was performed at week 10 with the same dose (*n* = 12). Mice from control groups were injected with PBS (*n* = 6). Mice were euthanized at week 15 to examine drug effects. One experiment was performed for IHC analysis and two independent experiments were performed to analyze ileal gene expression (one comprising GF Zeb2-transgenic mice).

### Tumor immune profiling by immunohistochemistry in the Zeb2-transgenic model

Immunohistochemistry staining, scanning, and analysis of double-staining CD8–CD3. Rabbit anti-mouse CD8α monoclonal Ab (Cell signaling, #D4W2Z, 1:400) and rabbit anti-mouse CD3 polyclonal Ab (Dako, #IS503) were performed on 3-µm-thick sections of formalin-fixed, paraffin-embedded colon from murine samples (nontreated mice, *n* = 6, and treated mice, *n* = 12). Antigen retrieval was performed by incubating slides in Tris-EDTA (pH 8.0) for 30 min at 98 °C. Then the antibodies were successively incubated for 1 h for CD8 and CD3 at room temperature, and detected respectively by PowerVision poly HRP anti-rabbit IgG (MM France, #PV6119). These slides were revealed by DiAminoBenzidine (DAKO, #K3468). Finally, the sections are counterstained by Hematoxylin (RAL Diagnostics, #3205501000). Images displayed in the figures were acquired as WSIs with a slide scanner Olympus VS120 at 20× objectives. For mouse tissue analysis, QuPath software was used [37]. Regions of interest (ROIs) of normal tissue or tumor were defined by two pathologists (SY and PO). CD3^+^ or CD8^+^ cells were identified using QuPath software.

### FMT experiments

Tumor growth and FACS analysis of tdLN were reported before [5].

### Subcutaneous model of MC38 for therapy settings

MC38 cell line was derived from methylcholanthrene-induced murine colon adenocarcinoma cells (NCI, Bethesda, MD) and tested negative for mycoplasma contamination. Syngeneic C57BL/6J mice were implanted with 1 × 10^6^ MC38 WT cells s.c. and treated i.p. when tumors reached 20–30 mm^2^ in size with 10 mg/kg OXA or vehicle (PBS). Mice of the same age and gender were randomly allocated in the different experimental groups, ensuring an equal distribution of tumor sizes for therapeutic experiments.

### Gut colonization with dedicated species

The tested species, *Bacteroides fragilis* and *Paraprevotella clara*, were isolated by culturomics from ileum samples from a previous study [5]. Species were grown on 5% sheep blood-enriched Columbia agar (bioMerieux) in an anaerobic atmosphere created using anaerobic generators (bioMerieux) at 37 °C for 24–72 h. Colonization of MC38-bearing SPF mice was performed by oral gavage with each species (*n* = 6 mice per group). For bacterial gavage, colonies were resuspended in PBS at a concentration of 10^9^ CFU/mL, evaluated using a fluorescence spectrophotometer (Eppendorf) at an optical density of 1 measured at a wavelength of 600 nm. Two bacterial gavages were performed for each mouse, the first, 24 h before the treatment with OXA and then 24 h after the treatment.

### Flow cytometry analyses

Tumors and ileal cells were harvested at the end of the experiment for immunological analysis. Gut dissociation to harvest cells from the epithelial compartment was performed as previously described [38]. Briefly, ilea and/or colons were collected and fat tissue, Peyer’s patches, and feces were removed. Intestines were cut longitudinally and then cut transversally into small pieces into a tube. Pieces were transferred into a new 50-mL tube with 20 mL of dissociation medium (PBS, 5% FCS, 5 mM EDTA, and 1 mM DTT), vortexed, and shaken at 37 °C for 15 min. Cell suspensions were collected in a new tube, filtered with a cell strainer (100 μm), centrifuged, and resuspended in PBS. The dissociation steps were performed twice and cell suspensions stored on ice until use. For tumor dissociation, tumors were cut into pieces of 2-mm side in RPMI medium, digested with Liberase™ TM (Sigma) and DNAse I (Roche) for 30 min at 37 °C. Subsequently, the cells were meshed through a 100-μm cell strainer to retrieve single-cell suspensions. In all cases, two million cells were preincubated with purified anti-mouse CD16/CD32 (eBioscience, catalog reference 16-0161-86) for 20 min at 4 °C, before membrane staining. Dead cells were excluded using the Live/Dead Fixable Yellow dead cell stain kit (Life Technologies). For cell phenotyping, the following antibodies were purchased from BD Pharmingen: anti-mouse mouse CD45 (catalog reference: 560510), anti-mouse CXCR5 (catalog reference: 551960), and anti-mouse CD3e (catalog reference: 552774). The following antibodies were purchased from BioLegend: anti-mouse CD4 (catalog reference: 100428), anti-mouse CXCR3 (catalog reference: 126531). The following antibodies were purchased from R&D systems: anti-mouse CCR6 (catalog reference: FAB590P). The following antibody was purchased from Invitrogen: anti-mouse PD-1 (catalog reference: 17-9985-82). Samples were acquired on 13-color Cytoflex (Beckman Coulter) and analyses were performed with FlowJo v10 software (Tree Star, Ashland, OR, USA).

### External data availability of 16S RNA seq

Microbiota characterization was already reported [5]. All raw sequencing data can be found at the NCBI Sequence Read Archive (accession number: PRJNA478491; “Ileal Apoptosis and Microbiome Shape Immunosurveillance and Prognosis of Proximal Colon Cancer”, https://www.ncbi.nlm.nih.gov/bioproject/?term=PRJNA478491, or https://www.ncbi.nlm.nih.gov/sra/PRJNA478491).

### Statistical analysis

The Mann–Whitney *U* test was used in comparisons of two group parameters, after Kruskal–Wallis test for multiple groups. The false-discovery rate was tested by the Benjamini–Hochberg procedure [39]. Survival curves were estimated using the Kaplan–Meier product limit method. Association between variables and outcome was evaluated using the log-rank test. Hierarchical clustering has been done with the distance 1-Pearson correlation coefficient and Ward’s agglomeration method. Statistics and graphics were performed using the R software and GraphPad Prism v7.03. All tests were two-sided, with mean ± SEM depicted, and *P* values  < 0.05 were considered statistically significant.

For human sample studies, the investigators were blinded to group allocation during data collection and analysis. For mice experiments, the investigators were not blinded to group allocation when performing treatments during the experiment, but they were blinded during data collection of outcome and analysis.

## Supplementary information

Supplemental Figure 1

Supplemental Figure 2

Supplemental Figure 3

Supplemental Figure 4

Supplemental Tables
