# Supplementary figures and images for: Ileal immune tonus is a prognosis marker of proximal colon cancer in mice and patients
Source: Cell Death Differ. 2020 Dec 1;28(5):1532–47. doi: 10.1038/s41418-020-00684-w (PMC8167112; doi:10.1038/s41418-020-00684-w)

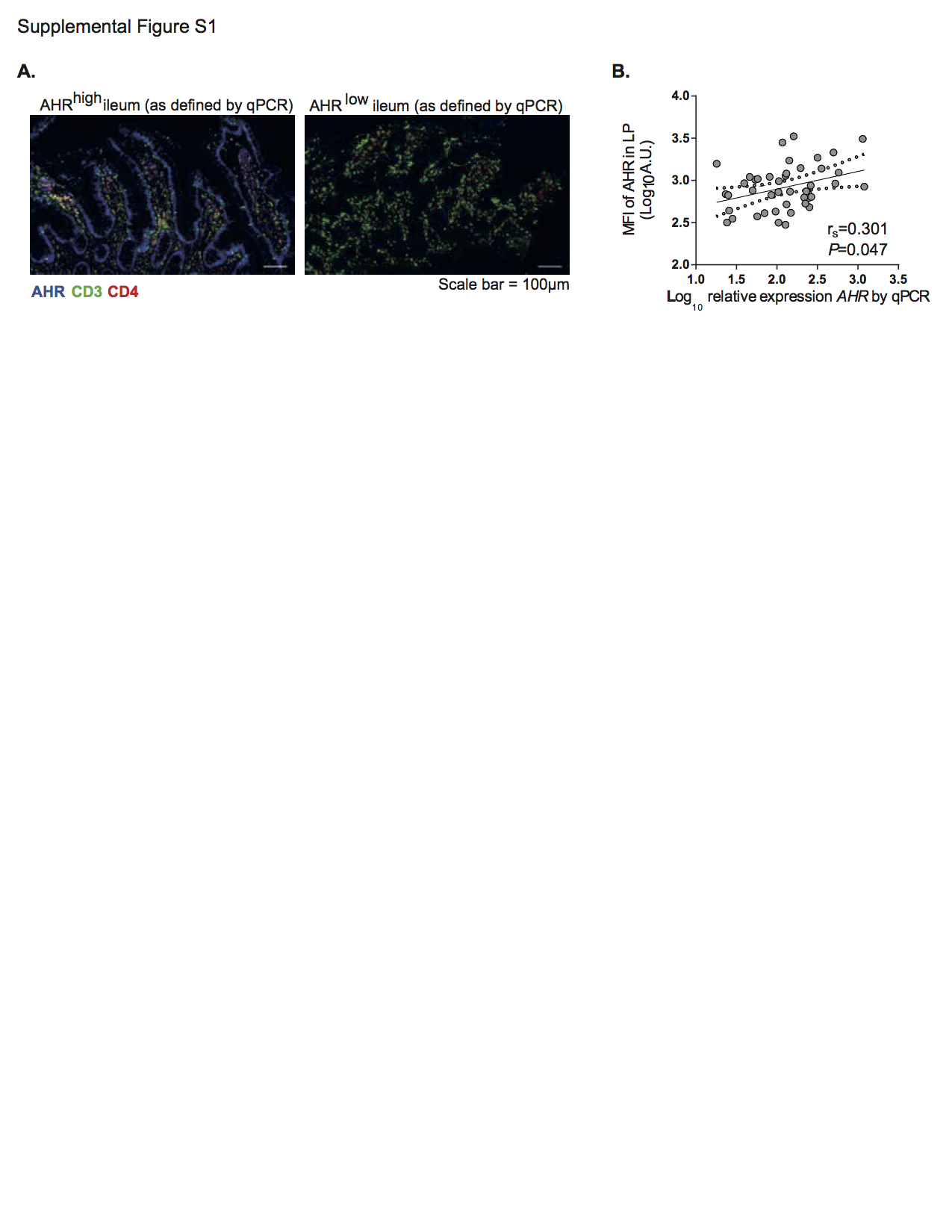

Supplement: Supplementary file 1 — Supplemental Figure 1 [file 41418_2020_684_MOESM1_ESM.tif]

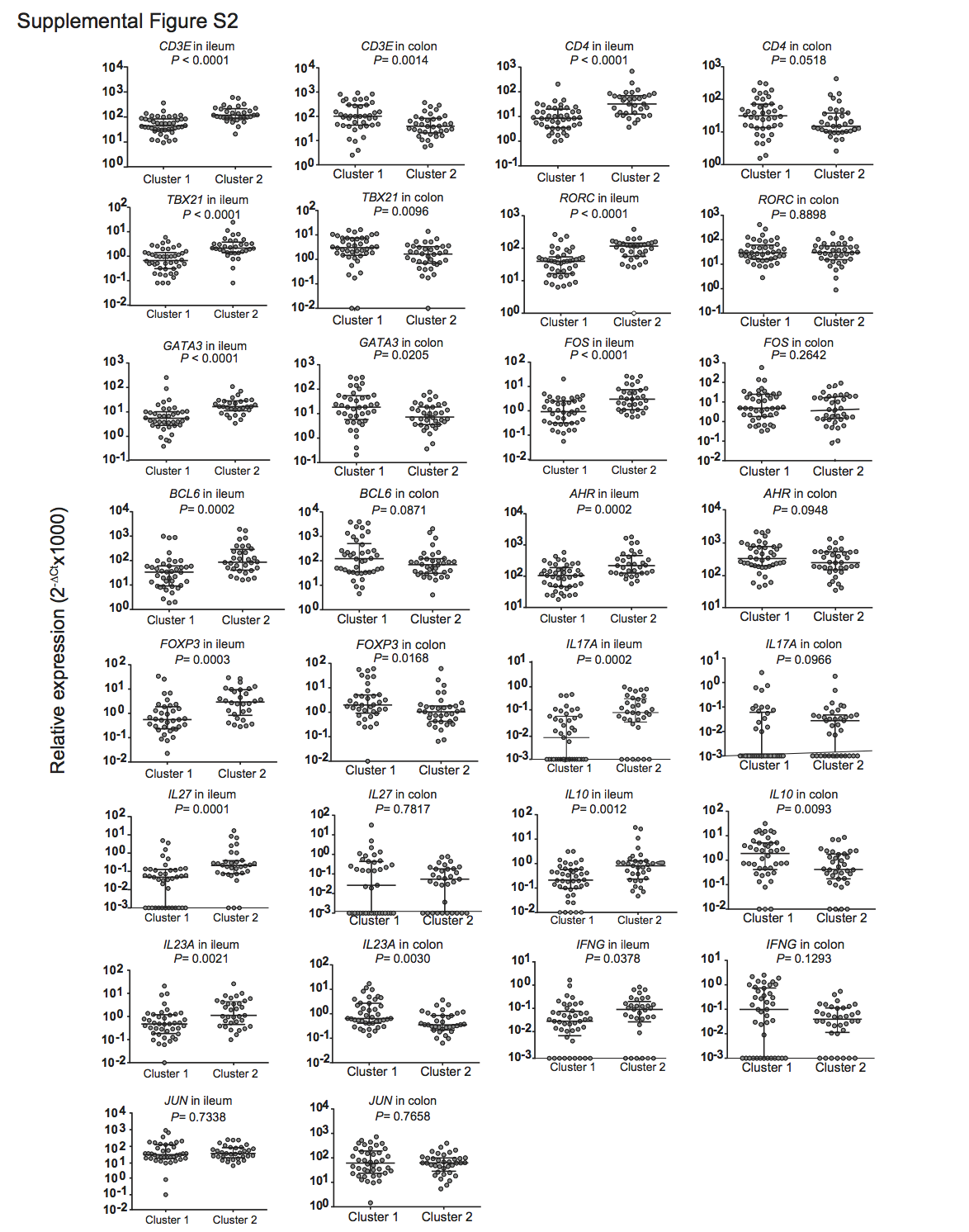

Supplement: Supplementary file 2 — Supplemental Figure 2 [file 41418_2020_684_MOESM2_ESM.tif]

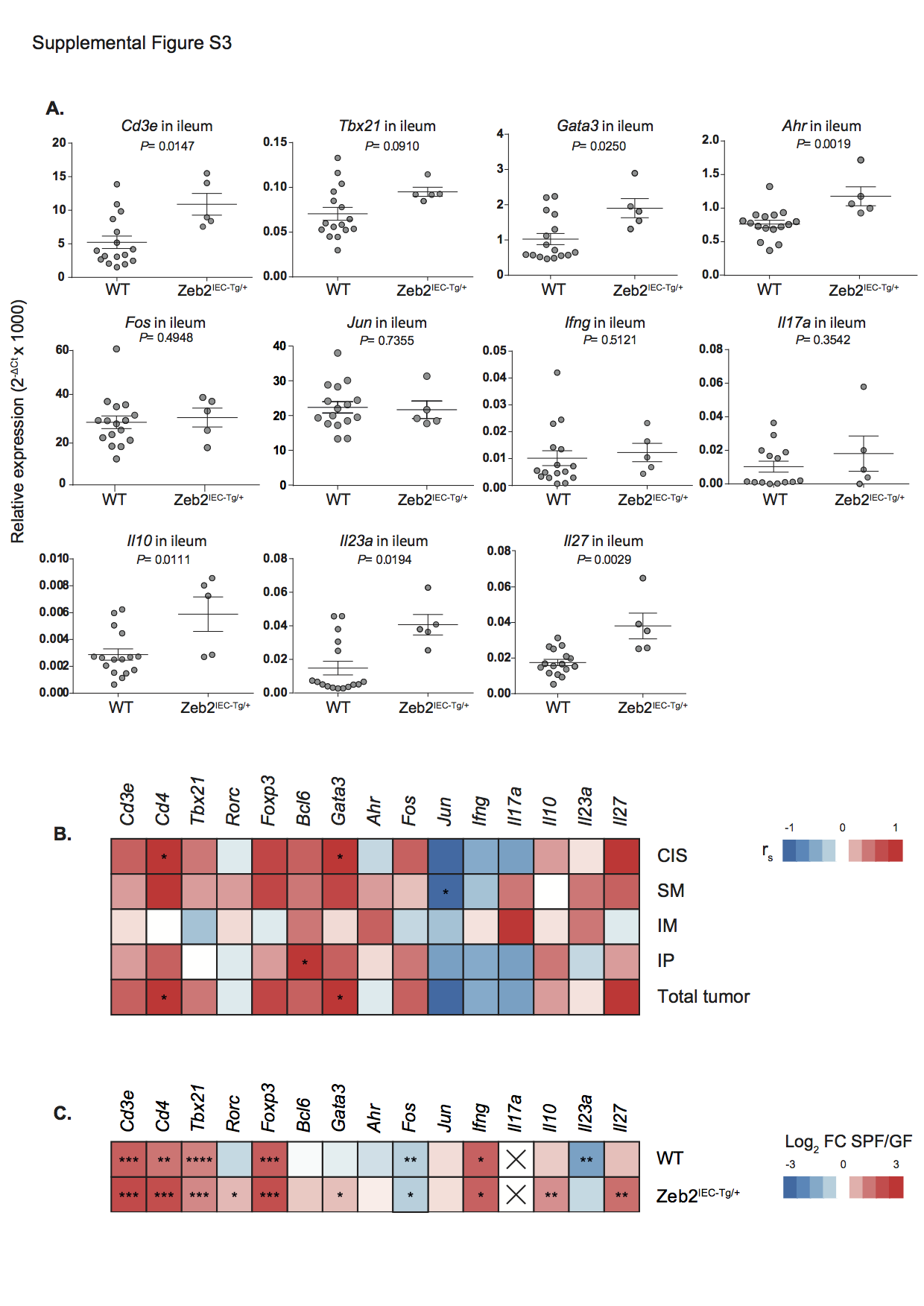

Supplement: Supplementary file 3 — Supplemental Figure 3 [file 41418_2020_684_MOESM3_ESM.tif]

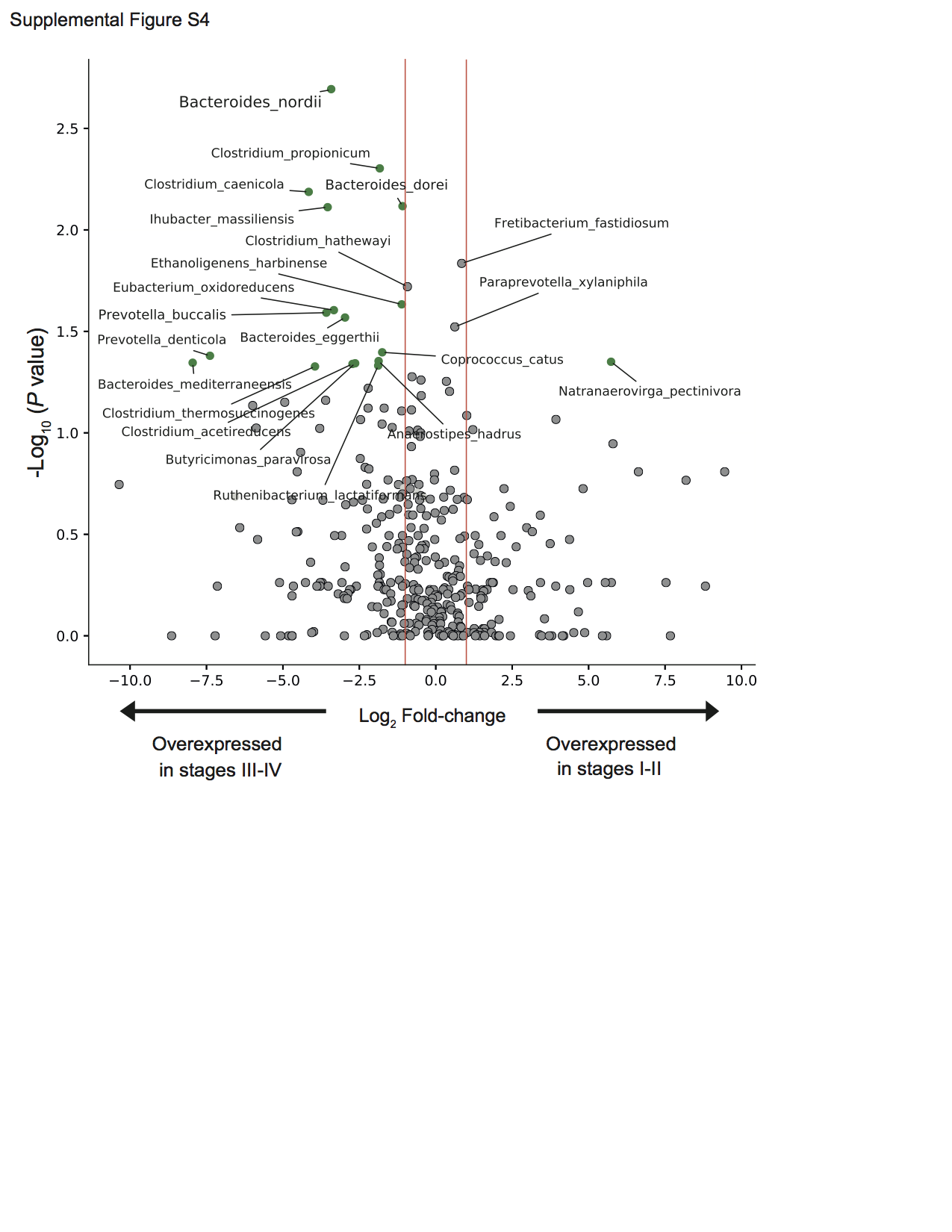

Supplement: Supplementary file 4 — Supplemental Figure 4 [file 41418_2020_684_MOESM4_ESM.tif]
